# Repeat pulmonary resection for lung malignancies does not affect the postoperative complications: a retrospective study

**DOI:** 10.1186/s12890-021-01477-8

**Published:** 2021-04-01

**Authors:** Nozomu Motono, Shun Iwai, Yoshihito Iijima, Katsuo Usuda, Hidetaka Uramoto

**Affiliations:** Department of Thoracic Surgery, Kanazawa Medical University, 1-1 Daigaku, Uchinada, Ishikawa 920-0293 Japan

**Keywords:** Repeat pulmonary resection, Multiple lung cancer, Postoperative complication, Video-assisted thoracic surgery

## Abstract

**Background:**

Although repeat pulmonary resection (RPR) for multiple lung cancer has been performed for non-small cell lung cancer and metastatic lung tumor, with the prognostic benefit detailed in several reports, the risk of RPR has not been well analyzed.

**Methods:**

Patients with lung malignancies who underwent complete resection at Kanazawa Medical University between January 2010 and October 2019 were analyzed. The relationship between postoperative complications and preoperative and perioperative factors was analyzed. Postoperative complications were categorized into five grades according to the Clavien–Dindo classification system.

**Results:**

A total of 41 patients who were received RPR were enrolled in this study. Primary lung tumor was found in 31 patients, and metastatic lung tumor was found in 10 patients. The postoperative complication rate of the first operation was 29%, and that of the second operation was 29%. While there were no significant factors for an increased incidence of postoperative complication in a multivariate analysis, an operation time over 2 h at the second operation tended to affect the incidence of postoperative complication (*p* = 0.06). Furthermore, the operation time was significantly longer (*p* = 0.02) and wound length tended to be longer (*p* = 0.07) in the ipsilateral group than in the contralateral group. The rate of postoperative complications and the length of the postoperative hospital stay were not significantly different between the two groups.

**Conclusion:**

RPR is safely feasible and is not associated with an increased rate of postoperative complications, even on the ipsilateral side.

## Background

Repeat pulmonary resection (RPR) for multiple lung cancer (MLC) has been performed for non-small cell lung cancer (NSCLC) and metastatic lung tumor, and the prognostic benefit has been described in several reports [1–9]. However, the risk of RPR has not been well analyzed. Although the mortality for patients who have undergone RPR was reported to range from 5 to 11% in previous reports [10–14], the mortality and complication rate for RPR have evolved thanks to recent improvements in surgical procedures.

Video-assisted thoracic surgery (VATS) for NSCLC patients has been widely adopted and the benefits of this approach have been reported [15–20]. Previous studies have shown that VATS is less painful, has a shorter hospital stay, has less reduced inflammatory-immune response, and is associated with postoperative maintenance of respiratory function compared with thoracotomy [17–19]. However, the relationship between the VATS approach and the rate of postoperative complications is not clear.

In the present study, we evaluated the perioperative variables and the risk of RPR in MLC patients.

## Methods

### Patients

Eight hundred and eighty-eight patients with lung malignancies who underwent complete resection in Kanazawa Medical University between January 2010 and October 2019 were identified. Among these, 468 NSCLC patients and 10 metastatic lung tumor patients had available data. Forty-one patients underwent repeat pulmonary resection, and 437 patients underwent single pulmonary resection. These patients were enrolled in the present retrospective study and analyzed.

About collected data, the clinical factors included the sex, age, smoking history, comorbidities, and the carcinoembryonic antigen (CEA), vital capacity as percent of predicted (%VC), forced expiratory volume in 1 second percent as percent of forced vital capacity (FEV1%), the side of lung tumor, and the diagnosis of lung tumor. The smoking history was evaluated using the Brinkman index, which is calculated as the number of cigarettes smoked per day multiplied by the number of years for which the subject has smoked [21]. The perioperative factors included the wound length, operative approach, operative procedure, and operation time. The operative approach was divided into three categories: complete VATS (C-VATS; surgery was only performed to provide a monitoring view); hybrid VATS (H-VATS; surgery was combined with direct vision without rib spreading); and thoracotomy. Postoperative complications were categorized into five grades according to the Clavien–Dindo classification system [22].

The present study was conducted in accordance with the amended Declaration of Helsinki. The Institutional Review Boards of Kanazawa Medical University approved the protocol (approval number: I392), and written informed consent was obtained from all of the patients. All data was anonymised before its use.

### Statistical analyses

The cumulative survival rates were calculated by the Kaplan–Meier method, and survival curves were compared using a log-rank test. Multivariate analyses using a stepwise logistic regression model was conducted to determine the risk factors for postoperative complication. All statistical analyses were two-sided, and *p* values of < 0.05 were considered to indicate statistical significance. The statistical analyses were conducted using the JMP software program (Version 13.2; SAS Institute Inc., Cary, NC, USA).

The present study was conducted in accordance with the amended Declaration of Helsinki. The Institutional Review Boards of Kanazawa Medical University approved the protocol (approval number: I392), and written informed consent was obtained from all of the patients.

## Results

### Patient characteristics

The clinicopathological characteristics of the 41 patients who received RPR in the present study are listed in Table 1. The median follow-up time was 1457 days (range: 162 to 5758 days). Thirty-one patients were men, and the median age at the first operation was 68 years old, while that at the second operation was 70 years old. The median Brinkman index was 160, and ipsilateral resection was performed in 13 patients. Primary lung tumor was found in 31 patients and metastatic lung tumor in 10 patients. The median %VC at the first operation was 102.9%, and that at the second operation was 87.4%. The median FEV1% at the first operation was 73.3%, and that at the second operation was 70.3%.

**Table 1:** Patient characteristics

1st age (years)	68 (37–80)
2nd age (years)	70 (37–81)
Gender (male/female)	31/10
Smoking index	160 (0–2000)
Ipsilateral/contralateral	13/28
R → R	12
R → L	17
L → L	2
L → R	10
Primary/metastatic	31/10
1st %VC	102.9 (63.1–140)
1st FEV_1.0_%	73.3 (40.8–98)
1st approach (C/H/T)	11/29/1
1st operative procedure (Part/Seg/Lob)	17/1/23
1st wound length (mm)	7 (3–20)
1st operation time (min)	166 (46–580)
1st postoperative complication (present/absent)	12/29
1st Clavien–Dindo grade (0/1/2/3a)	29/1/5/6
2nd %VC	87.4 (60.5–122.7)
2nd FEV_1.0_%	70.3 (43.1–86)
2nd approach(C/H/T)	14/23/4
∆%VC	− 12 (− 42.2–5.8)
∆FEV_1.0_%	− 2.6 (− 30.1–17.2)
2nd operative procedure (Part/Seg/Lob)	27/7/7
2nd wound length (mm)	6 (3–20)
2nd operation time	126 (46–501)
2nd postoperative complication (present/absent)	12/29
2nd Clavien–Dindo grade (0/1/2/3a)	29/0/4/8
1st postoperative hospital stay (days)	15 (4–36)
2nd postoperative hospital stay (days)	12 (4–62)
Interval from 1st to 2nd operation (days)	406 (28–4529)

%VC, predictive vital capacity; FEV_1.0_%, forced expiratory volume in 1 second/forced vital capacity ratio; C, complete video-assisted thoracic surgery; H, hybrid video-assisted thoracic surgery; T, thoracotomy; Part, partial resection; Seg, segmentectomy; Lob, lobectomy; ∆%VC, rate of 2nd %VC per 1st %VC; ∆FEV_1.0_%, rate of 2nd FEV_1.0_% per 1st FEV_1.0_%

### Perioperative factors

The first operative approach was C-VATS in 11 patients, H-VATS in 29, and thoracotomy in 1. The second operative approach was C-VATS in 14 patients, H-VATS in 23, and thoracotomy in 4. The median wound length of the first operation was 7 mm, and the median operation time was 166 min. The median wound length of the second operation was 6 mm, and the median operation time was 126 min. Sublobar resection in 18 patients and lobectomy in 23 was performed at the first operation. Sublobar resection in 34 patients and lobectomy in 7 was performed at the second operation. The postoperative complication rate of the first operation was 29%, and the postoperative complications were classified as Clavien–Dindo grade 0 in 29 patients, grade I in 1, grade II in 5, and grade IIIa in 6. All six patients with grade IIIa complications had prolonged air leakage and underwent pleurodesis. The postoperative complication rate of the second operation was 29%, and the postoperative complications were classified as Clavien–Dindo grade 0 in 29 patients, grade I in 0, grade II in 4, and grade IIIa in 8. All eight patients with grade IIIa complications had prolonged air leakage and underwent pleurodesis. The incidence of postoperative complications in the RPR and single pulmonary resection (SPR) groups was not significantly different (data not shown; RPR: SPR = 29%:26%, *p* = 0.71). The median postoperative hospital stay after the first operation was 15 days, while that after the second operation was 12 days.

### Bivariate analyses

The relationship between patients’ characteristics and perioperative factors and postoperative complications after RPR is shown in Table 2. Although the gender, age, smoking history, operative side, operative procedure, %VC, FEV1%, approach at the second operation, and duration of the second operation were analyzed, these factors did not significantly affect the incidence of postoperative complications.

**Table 2:** Bivariate analysis of relationship between patients’ characteristics and perioperative factors and postoperative complication

	Complication % (n)	*p* value
Gender		
Male	29 (9/31)	0.95
Female	30 (3/10)	
Age		
< 70 years	22 (4/18)	0.38
≧ 70 years	35 (8/23)	
Smoking history		
Never	20 (4/20)	0.21
Current/former	38 (8/21)	
Side		
Ipsilateral	31 (4/13)	0.88
Contralateral	29 (8/28)	
1st operative procedure		
Part	29 (5/17)	0.98
Seg/Lob	29 (7/24)	
1st postoperative complication		
Absent	31 (9/29)	0.69
Present	25 (3/12)	
2nd %VC		
< 80	20 (3/15)	0.69
≧ 80	34 (9/26)	
2nd FEV_1.0_%		
< 70	25 (5/20)	0.56
≧ 70	33 (7/21)	
2nd approach		
C	29 (8/27)	0.94
H/T	28 (4/14)	
2nd operative procedure		
Part	29 (8/27)	0.94
Seg/Lob	28 (4/14)	
2nd operation time		
< 2 h	20 (4/20)	0.67
≧ 2 h	38 (8/21)	

Part, partial resection; Seg, segmentectomy; Lob, lobectomy; %VC, predictive vital capacity; FEV_1.0_%, forced expiratory volume in 1 second/forced vital capacity ratio; C, complete video-assisted thoracic surgery; H, hybrid video-assisted thoracic surgery; T, thoracotomy

### Multivariate analyses

Our multivariate analysis of postoperative complications examined the joint effects of the gender, age, smoking history, operative side, procedure for the first operation, postoperative complications after the first operation, %VC at the second operation, FEV1% at the second operation, approach at the second operation, procedure for the second operation, and duration of the second operation. There were no significant factors affecting the incidence of postoperative complications (Table 3).

**Table 3:** Multivariate analysis for postoperative complication

	OR	95% CI	*p* value
Gender (male)	1.15	0.17–7.65	0.87
Age (≧ 70 years)	1.67	0.23–12.01	0.61
Smoking history (Curr + Former)	4.27	0.66–27.58	0.12
Side (ipsilateral)	1.08	0.19–6.13	0.92
1st Operative procedure (Seg + Lob)	1.92	0.25–14.74	0.52
1st Postoperative complication (present)	0.36	0.04–3.07	0.35
2nd %VC (< 80%)	0.53	0.08–3.35	0.50
2nd FEV_1.0_% (< 70%)	0.22	0.03–1.68	0.14
2nd approach (H + T)	0.52	0.06–4.03	0.53
2nd operative procedure (Seg + Lob)	0.28	0.02–2.74	0.27
2nd operation time (≧ 2 h)	12.65	0.86–184.77	0.06

Curr, current; %VC, predictive vital capacity; FEV_1.0_%, forced expiratory volume in 1 second/forced vital capacity ratio; Seg, segmentectomy; Lob, lobectomy H, hybrid video-assisted thoracic surgery; T, thoracotomy,

### Sub-analyses

The relationship between the operative side (ipsilateral or contralateral) and perioperative factors was analyzed (Table 4). The operation time was significantly longer (*p* = 0.02), and the wound length tended to be longer (*p* = 0.07) in the ipsilateral group than in the contralateral group. The rate of postoperative complications and the length of the postoperative hospital stay were not significantly different between the two groups.

**Table 4:** Sub-analysis of relationship between operative side and perioperative factors

	Ipsilateral	Contralateral	*p* value
2nd operation time	151 (68–315)	110 (46–501)	0.02
2nd postoperative complication (present/absent)	4/9	8/20	0.88
2nd wound length (mm)	8 (4–20)	5 (3–15)	0.07
2nd approach (C/H/T)	3/7/3	11/16/1	0.12
2nd operative procedure(Part/Seg/Lob)	8/2/3	19/5/4	0.78
2nd postoperative hospital stay (days)	12 (4–62)	12 (4–27)	0.98

C, complete video-assisted thoracic surgery; H, hybrid video-assisted thoracic surgery; T, thoracotomy; Part, partial resection; Seg, segmentectomy; Lob, lobectomy

### Survival analyses

**Fig. 1:**
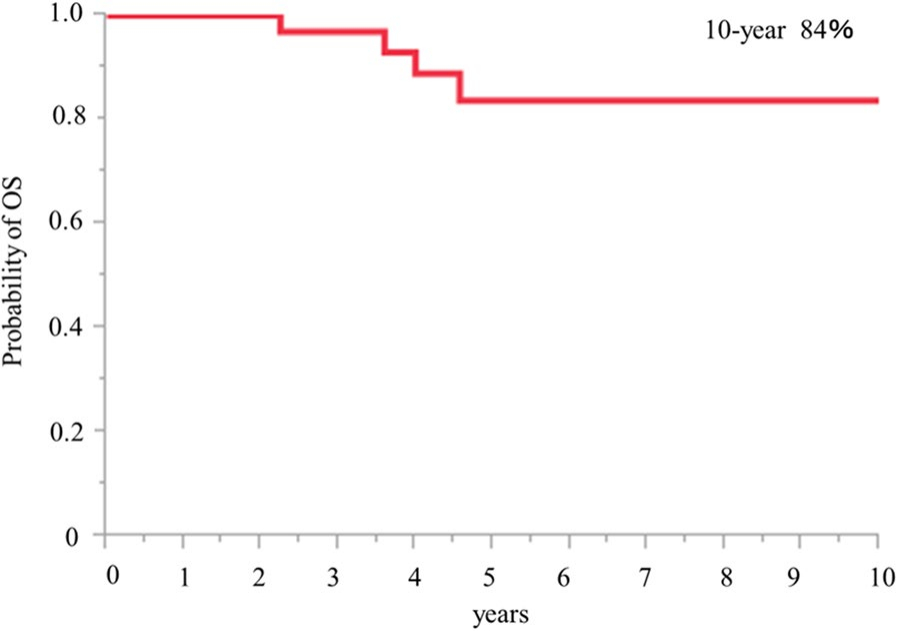
Overall survival after 1st operation is shown. 10-year survival rate is 84%

**Fig. 2:**
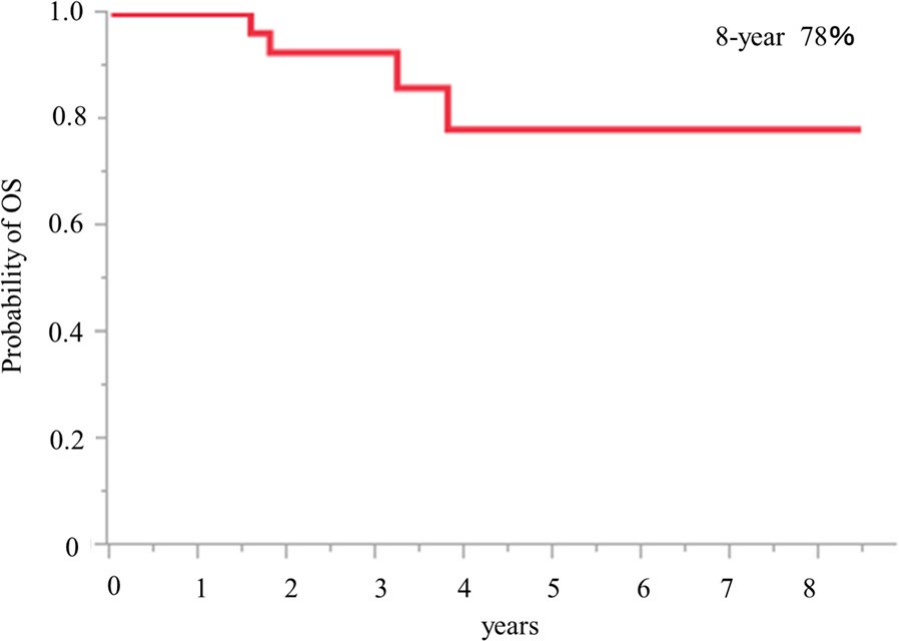
Overall survival after 2nd operation is shown. 8-year survival rate is 78%

The overall survival from the first operation is shown in Fig. 1. The 10-year overall survival was 84%. The overall survival from the second operation is shown in Fig. 2. The 8-year overall survival was 78%.

## Discussion

We evaluated the risk of RPR for MLC in the present study. RPR was not found to be associated with the risk of postoperative complications, and there was no perioperative mortality. The incidence of postoperative complications with RPR was reported to range from 19 to 33% in previous studies [1, 3, 4, 9, 10]. Although the incidence of postoperative complications at the second operation in the present study was 29%, all patients with postoperative complications of grade IIIa had air leakage that required pleurodesis. The rate of other complications classified as grades I and II was 14% at the first operation and 9% at the second operation. These findings indicate that severe postoperative complications were relatively rare. The VATS approach for managing lung malignancies has been widely adopted and is reported to be less invasive than thoracotomy [15–20]. Because both the first and second operations were performed via the VATS approach in most cases in the present study, it might have been affected less invasive and physical function maintenance.

In previous studies, the mortality separated by operative procedure was reported; the mortality rate was 34% for pneumonectomy, 7% for lobectomy, 0% for segmentectomy, and 6% for partial resection [10]. The mortality rate might have been lower than in previous studies because there were more cases of partial resection and segmentectomy than lobectomy at the second operation in the present study. Although cases of sublobar resection accounted for more than 80% for second operations, the 8-year overall survival was 78%, suggesting that sublobar resection might have a good prognosis. Furthermore, sublobar resection might maintain the respiratory function and enable a third round of pulmonary resection.

RPR has diagnostic and therapeutic implications. The prevalence of a second malignancy in lung cancer patients investigated in previous studies was reported 1.87–2.41% [23, 24]. Although a histological examination is necessary for cases of metastasis or second primary lung cancer, the histological pattern may have changed in relapse tumors. Molecular assessments, such as gene array analyses or patterns of loss of heterozygosity, are useful for the identification of the independence of lung primaries, underscoring the importance of obtaining acceptable specimens. Furthermore, favorable outcomes were reported for select stage IV NSCLC patients who received complete resection of both the primary lung tumor and metastasis, such as solitary adrenal gland, brain or contralateral lung metastasis [25–27].

A previous study showed that an operation time exceeding two hours was a predictor of postoperative complications [10]. In the present study, an operation time exceeding two hours tended to increase the risk of postoperative complications in our multivariate analysis. Although ipsilateral resection for MLC had a significantly longer operation time than contralateral resection, the incidence of postoperative complications was not significantly different between ipsilateral and contralateral resection (*p* = 0.88). Therefore, ipsilateral RPR itself might be not predictor of postoperative complications.

The present study was associated with several limitations. First, the study was retrospective in nature and potentially involved unobserved cofounding and selection biases. Second, our study was performed at a single institution, and the study population was relatively small.

## Conclusions

RPR is safely feasible and is not associated with an increased rate of postoperative complications, even on the ipsilateral side. RPR might improve the prognosis, and patient selection is important.

## Data Availability

The datasets used and/or analysed during the current study are available from the corresponding author on reasonable request.

## Abbreviations

RPR: Repeat pulmonary resection
MLC: Multiple lung cancer
NSCLC: Non-small cell lung cancer
VATS: Video-assisted thoracic surgery
CEA: Carcinoembryonic antigen
%VC: Vital capacity as percent of predicted
FEV1%: Forced expiratory volume in 1 second percent as percent of forced vital capacity
C-VATS: Complete video-assisted thoracic surgery
H-VATS: Hybrid video-assisted thoracic surgery
SPR: Single pulmonary resection
